# FAP-overexpressing fibroblasts produce an extracellular matrix that enhances invasive velocity and directionality of pancreatic cancer cells

**DOI:** 10.1186/1471-2407-11-245

**Published:** 2011-06-13

**Authors:** Hyung-Ok Lee, Stefanie R Mullins, Janusz Franco-Barraza, Matthildi Valianou, Edna Cukierman, Jonathan D Cheng

**Affiliations:** 1 Department of Medical Oncology, Fox Chase Cancer Center, 333 Cottman Avenue, Philadelphia, Pennsylvania 19111, USA; 2 Cancer Biology Program, Fox Chase Cancer Center, 333 Cottman Avenue, Philadelphia, Pennsylvania 19111, USA

## Abstract

**Background:**

Alterations towards a permissive stromal microenvironment provide important cues for tumor growth, invasion, and metastasis. In this study, Fibroblast activation protein (FAP), a serine protease selectively produced by tumor-associated fibroblasts in over 90% of epithelial tumors, was used as a platform for studying tumor-stromal interactions.

We tested the hypothesis that FAP enzymatic activity locally modifies stromal ECM (extracellular matrix) components thus facilitating the formation of a permissive microenvironment promoting tumor invasion in human pancreatic cancer.

**Methods:**

We generated a tetracycline-inducible FAP overexpressing fibroblastic cell line to synthesize an *in vivo*-like 3-dimensional (3D) matrix system which was utilized as a stromal landscape for studying matrix-induced cancer cell behaviors. A FAP-dependent topographical and compositional alteration of the ECM was characterized by measuring the relative orientation angles of fibronectin fibers and by Western blot analyses. The role of FAP in the matrix-induced permissive tumor behavior was assessed in Panc-1 cells in assorted matrices by time-lapse acquisition assays. Also, FAP^+ ^matrix-induced regulatory molecules in cancer cells were determined by Western blot analyses.

**Results:**

We observed that FAP remodels the ECM through modulating protein levels, as well as through increasing levels of fibronectin and collagen fiber organization. FAP-dependent architectural/compositional alterations of the ECM promote tumor invasion along characteristic parallel fiber orientations, as demonstrated by enhanced directionality and velocity of pancreatic cancer cells on FAP^+ ^matrices. This phenotype can be reversed by inhibition of FAP enzymatic activity during matrix production resulting in the disorganization of the ECM and impeded tumor invasion. We also report that the FAP^+^ matrix-induced tumor invasion phenotype is β_1_-integrin/FAK mediated.

**Conclusion:**

Cancer cell invasiveness can be affected by alterations in the tumor microenvironment. Disruption of FAP activity and β_1_-integrins may abrogate the invasive capabilities of pancreatic and other tumors by disrupting the FAP-directed organization of stromal ECM and blocking β_1_-integrin dependent cell-matrix interactions. This provides a novel preclinical rationale for therapeutics aimed at interfering with the architectural organization of tumor-associated ECM. Better understanding of the stromal influences that fuel progressive tumorigenic behaviors may allow the effective future use of targeted therapeutics aimed at disrupting specific tumor-stromal interactions.

## Background

Increasing evidence demonstrates the significance of the tumor microenvironment for tumor initiation and progression [1-6]. The tumor microenvironment is characterized by a heterogeneous complex of cellular and acellular components including tumor-associated fibroblasts, immune and endothelial cells, soluble cytokines, chemokines and proteases, as well as a characteristically remodeled ECM [7]. These components act in a coordinated manner to regulate the growth and differentiation of adjacent cells, thus alterations in the stromal microenvironment towards a permissive environment provide important cues for tumor growth, invasion, and metastasis [7,8]. In fact, the majority of proteases and stromal factors associated with malignant tumors are secreted by the host stroma rather than by the tumor cells themselves [9-11]. One of the most selective proteins for tumor stromal fibroblasts is the Fibroblast Activation Protein (FAP).

FAP is a serine protease that contains both dipeptidyl peptidase and gelatinase/collagenage activities *in vitro *[12]. Because of its specific induction in tumor-associated fibroblasts in over 90% of epithelial tumors, including pancreas and breast among others, FAP was used as a platform for studying stromal specific effects on tumor behaviors [13-18]. Previously, we reported that FAP overexpression by tumor cells results in increased tumorigenicity and tumor growth [16] and its enzymatic activity played an important role in the promotion of tumor growth in mouse model [17]. Although several studies have shown that FAP expression in human melanoma cell lines or hepatic stellate cells promotes an invasive phenotype through cell adhesion pathways [13-15], it is not clear how FAP expressing fibroblast-specific signals prepare a permissive stromal microenvironment and how modified ECMs influence cancer cell behavior *in vitro*.

Studies in several human cancer types describing alterations in stromal cells and their ECM compositions suggest that changes in the microenvironment and tissue architecture contribute to tumorigenesis [19]. *In vivo*, the mesenchymal ECM is comprised of several proteins including collagens I, III and fibronectin, which assemble in an intricate fibrillar network and are engaged by transmembrane receptors. For example, integrins transmit biochemical and mechanical stimuli from the matrix to the cytoskeleton of the cell and back to the ECM, triggering distinct intracellular signaling pathways that control proliferation, survival, and migration [20]. In fact, it has been shown that both desmoplastic fibronectin and collagen I fibers often align in parallel patterns in response to tumorigenesis [21,22], and these fibers are engaged by integrin heterodimers in pancreatic and other cancers [6,20], suggesting that cell behavior can be affected by the underlying stromal substrates via integrin engagement. Thus, alteration of the stromal ECM composition in cancers may be linked to cancer progression through tissue remodeling processes [23]. Here, we show that FAP enzymatic activity locally modifies stromal ECM components thus facilitating the formation of a permissive microenvironment promoting tumor invasion on human pancreatic cancer.

## Methods

### Cell lines and murine xenograft model

All cell lines used in this study were originally purchased from ATCC or obtained from the Cell Culture Facility at Fox Chase Cancer Center. Three C.B17/Icr-*scid *mice were subcutaneously injected with 2 × 10^6 ^cells of each pancreatic cell line (Panc-1, Capan-1, AsPC-1, and HPAF-II). After 5 weeks inoculation, tumors were harvested for immunohistochemistry analysis.

### Stable transfection of fap in NIH-3T3 fibroblasts

Mouse *fap *gene was cloned downstream of the Tet-response promoter in a tetracycline-inducible expression vector pTRE (a kind gift from Dr. Teresa Ramirez-Montagut, Memorial Sloan-Kettering Cancer Center, NY). This construct was co-transfected into NIH-3T3 cells with rtTA plasmid (pUHD172-1 neo) that encodes the pTRE promoter-binding transactivator, thus murine FAP expression was induced by adding Doxycycline (Dox, 2 μg/ml) into complete DMEM medium containing 10% Tet system approved Fetal Bovine Serum (Clontech, CA).

### Isolation of human pancreatic stellate cells and matched pancreatic adenocarcinoma-associated fibroblasts

Fresh surgical tissue (decoded) samples from a pancreatic Whipple conducted at the Fox Chase Cancer Center was delivered with the assistance of the Protocol Laboratory and the Biosample Repository Facility following protocols approved by the Institutional Review Board. Tissue samples corresponding to pancreatic adenocarcinoma and distant (normal) pancreas were rinsed in cold PBS containing 100 μg/ml streptomycin and 100 U/ml penicillin. The two types of samples were carefully minced and incubated overnight with 0.2% collagenase at 37°C for digestive dissociation. The digested material was subjected to centrifugation at 1200 rpm for 5 min, thus precipitating a fibroblast-enriched fraction. This fraction was filtered through a series of 100 μm followed by 40 μm cell-strainer (BD Bioscience) before culturing in DMEM containing 10% FBS, 100 U/ml penicillin, 100 μg/ml streptomycin and 2 mM L-glutamine at 37°C using a humidified atmosphere and 5% CO_2_ for a period of 4 hours before removing the non adherent cells. Cell homogeneity was confirmed by direct microscopic observations, while cells were designated as fibroblastic following confirmation of mesenchymal marker vimentin expression, as well as absence of epithelial marker cytokeratin 19 expression. In addition, normal fibroblasts were characterized as pancreatic stellate cells following confirmation of uptake and storage of vitamin A, by auto-fluorescence stemming from retinyl acetate (Sigma) containing droplets [24]. The resultant (PaSCs) and pancreatic tumor-associated fibroblasts (which were confirmed to have lost the vitamin A droplets) were used for self-derived matrix characterization.

### Western analysis of FAP induction in fibroblasts

FAP-transfected fibroblasts were harvested at different time points (0, 1, 2, 4, 6, 8, and 10 days) in the presence or absence of Dox. Total protein was extracted using M-Per reagent (Pierce, IL), resolved by 4-12% SDS-PAGE under reducing conditions (Invitrogen, CA), and blotted with rabbit monoclonal anti-mouse FAP antibody (0.08 μg/ml). Immuno positive bands were labeled using goat anti-rabbit-HRP (3000×, Amersham Bioscience, UK) and visualized by ECL reagent (Pierce, IL). Purified murine recombinant FAP protein (92 kD) and parental NIH-3T3 cell lysate were used as positive and negative controls, respectively.

### Fibroblast-derived 3D matrix production

All fibroblasts were seeded at a concentration of 7 × 10^5 ^cells per sample onto 35 mm plates pre-coated with 0.2% gelatin. Confluent fibroblastic cultures were treated with media supplemented with 50 μg/ml ascorbic acid (and Dox when necessary) every other day for 8 days to obtain un-extracted 3D cultures. Alkaline detergent treatment (0.5% Triton X-100, 20 mM NH_4_OH in PBS) for 5 minutes at 37°C gave rise to cell-free *in vivo*-like 3D matrices [25,26]. For control, FAP-transfected fibroblasts were used to make FAP^- ^matrix in the absence of Dox. To make FAP+inhibitor matrix, FAP-specific small molecule inhibitor naphthalenecarboxy-Gly-boroPro (400 μM, provided by Dr. William Bachovchin, Tufts University, MA) was added to the media during matrix production.

### Analysis of fibronectin fiber orientation

For indirect immunofluorescent labeling of matrix fibers, un-extracted 3D cultures were prepared on glass cover slips as described [21]. Cells were stained with rabbit anti-mouse fibronectin antibody (25 μg/ml, Abcam, UK), a donkey anti-rabbit Cy5 conjugated antibody (15 μg/ml, Jackson ImmunoResearch, PA) and DAPI.

From two independent experiments with duplicate samples, minimum of 5 images per experimental sample were obtained using a z-stack function of the spinning disc microscope (PerkinElmer Life Sciences, PA). Each slice measured 0.5 μm and stacks were reconstituted as a maximum projection using the MetaMorph software as described in detail [21]. Fiber distributions corresponding to each experimental setting were determined by the percentage of fibers that were oriented at 10^0 ^variation angles from the identified modes.

### In-Cell Western analysis of un-extracted 3D cultures

FAP-transfected fibroblasts (1.5 × 10^4 ^cells/48-well plate) were cultured in the presence or absence of Dox during matrix production. Fibroblasts were fixed with 4% paraformaldehyde, permeabilized with 0.1% Triton X-100 in PBS, and stained with antibodies followed by manufacturer's directions (Li-Cor Bioscience, NE). The antibodies for this assay were tenascin C (50 μg/ml, Abcam, UK), α- SMA (5 μg/ml, Sigma, MO), collagen I (25 μg/ml, Chemicon International, CA), fibronectin (20 μg/ml, Abcam, UK), β-actin (1000×, Cell Signaling Technology, MA), and GAPDH (2 μg/ml, Chemicon International, CA). Fluorescence-labeled secondary antibodies IRDye 680 and IRDye800CW (Li-Cor Bioscience, NE) were used to scan by the Odyssey Infrared Imaging System. The protein levels were normalized by β-actin or GAPDH.

### Western analysis of pancreatic cancer cells cultured within assorted matrices

Following 2 days culture on matrices, Panc-1 cells were lysed in extraction buffer (50 mM Tris-HCl, pH8.8, 1% SDS, 5 mM DTT, 13 mM iodoacetamide, 5 mM EDTA, 150 mM NaCl). Proteins were quantified by western blot analysis using ECL reagent (Pierce, IL) or Odyssey infrared imaging system following the manufacturer's directions (Li-Cor Bioscience, NE). The antibodies for this assay were β1-integrin (2500×, BD Transduction Laboratories, NJ), AKT (500×, BD Transduction Laboratories, NJ), pS^473^-AKT (1000×, Cell Signaling Technology, MA), FAK (1 μg/ml, Upstate, NY), pY^397^-FAK (1000×, Biosource, CA), and GAPDH (2 μg/ml, Chemicon International, CA). Specific activity of AKT and FAK were calculated as the ratio of the scanned optical density of phosphorylated protein/total protein. Then the ratios were normalized by GAPDH.

### Motility assay within the assorted 3D matrices

As described in detail [26], tumor cells were re-plated onto the matrices and incubated overnight. Cell movements (10~15 cells), recording cells migrating both on and through the matrix, were recorded every 10 minutes during 12 hours using Nikon TE-2000U inverted microscope equipped with cool snap HQ camera. Individual cell dynamics were analyzed using the MetaMorph program following 4 distinctive factors: 1) the net path distance (D, μm) by calculating the number of μm/pixel. 2) The path trajectory of an individual cell during the recording period (T, μm). 3) Average velocity as the motility rate (AV, μm/hr). 4) Directionality calculated by the D/T ratio, which determines random (D/T = significantly smaller than 0.5) versus directional (D/T = close to 1) migration of individual cells [27-29]. Motility experiments using the assorted matrices were performed a minimum of 3 times using different batches of matrices and following a minimum of 10 individual cells.

In order to assess the effect on motility by integrin inhibitors, motility assays were performed in the presence of functional blocking β_1 _integrin antibody mAb13 (50 μg/ml, a kind gift from Dr. K. Yamada at NIH/NIDCR, Bethesda, MD) or the α_5_β_1_-integrin blocking peptide ATN-161 (Ac-PHSCN-NH2, 50 μg/ml, Attenuon, San Diego, CA). These experiments were performed at least 2 times using two alternative batches of matrices.

### Statistic analyses

Data from 3D matrix fiber distribution were analyzed using "chi-squared test". The normalized western data were analyzed using linear regression. For the motility analysis, the average velocity, net path distance and the path trajectory were analyzed using Gamma regression, and the directionality was analyzed using linear regression.

### Immunohistochemistry

Quick snap frozen tumor tissues sections (10 μm thick) from pancreatic cancer patients and xenografted mouse tumor tissues were incubated with rat monoclonal anti-human FAP antibody D8 (20×, a kind gift from Dr. Wen-Tien Chen, State University of New York, NY) and rabbit polyclonal anti-mouse FAP antibody (2 μg/ml) [16], respectively. Biotin-streptavidin detection with horseradish peroxidase (BioGenex, CA) was applied for the amplification and visualization of signals following the manufacturer's directions. Note that results stemming from this method are depicted in supplemental Figure 1.

**Figure 1:**
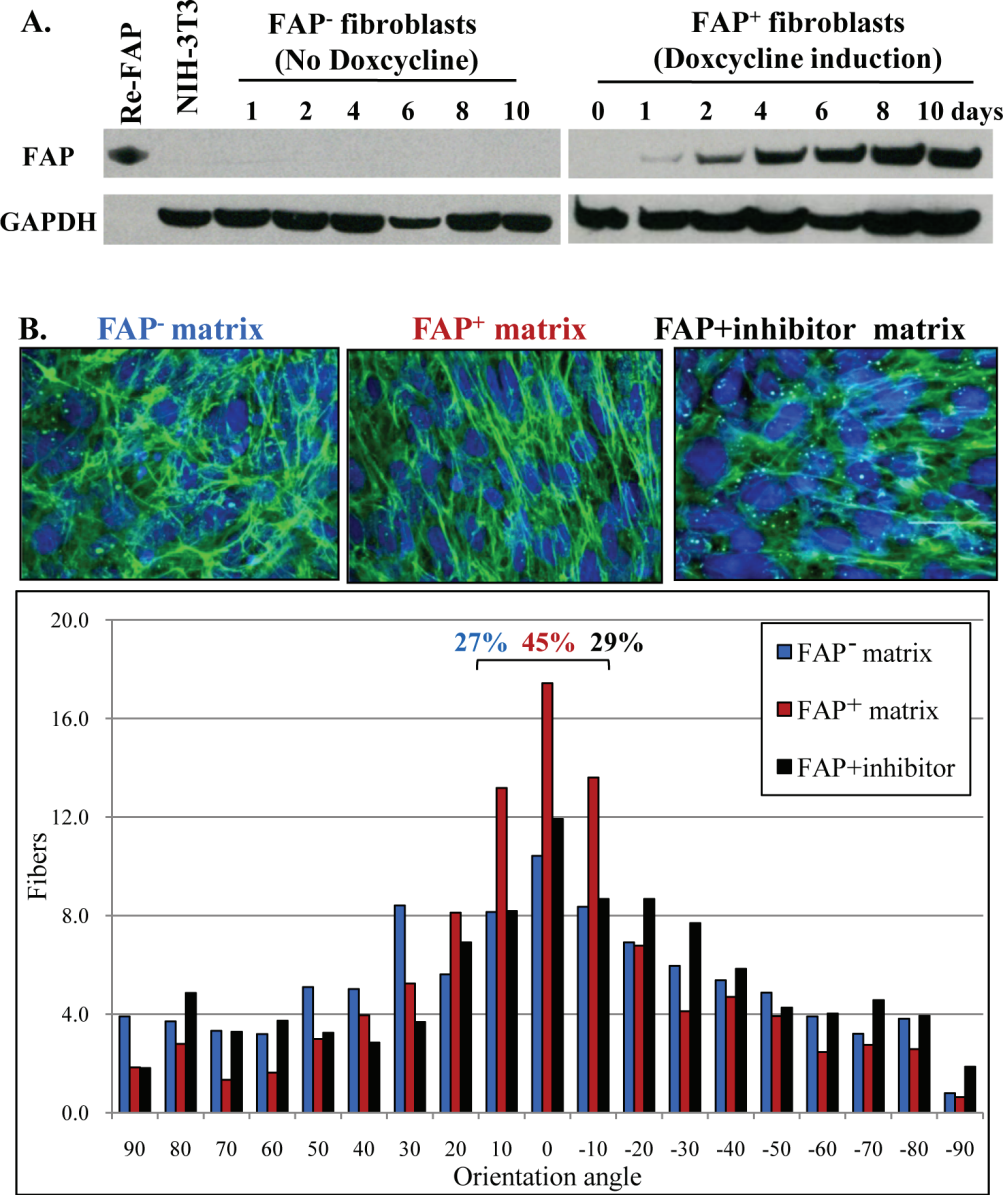
**Matrix fiber organization is increased in FAP^+ ^matrices**. (A) Western analysis showed that FAP was overexpressed in a Tet-inducible *fap*-transfected NIH-3T3 fibroblast as early as 24 hours following Dox treatment, and its expression maintained for at least 10 days. Parental NIH-3T3 cells and FAP^+ ^fibroblasts in the absence of Dox showed no detectable FAP induction. Recombinant FAP protein (Re-FAP, 92 kD) was used as positive control. (B) Un-extracted matrices were analyzed for the levels of fiber orientation by indirect immunofluorescence using a fibronectin antibody (green) and DAPI (blue). FAP^+ ^fibroblasts produced ECM fibers with enhanced parallel pattern compared to FAP^- ^and FAP+inhibitor matrices. The fiber distribution was determined by calculating the % of fibers arranged in parallel for each acquired region (± 10^0 ^of the mode angle). The observed % of fibers was 27%, 45%, and 29% for FAP^-^, FAP^+^, and FAP+inhibitor ECMs, respectively.

## Results

### *In vivo *tumor-dependent stromal FAP upregulation provides the rationale to study *in vitro *FAP fibroblastic over-expression effects

Some reports suggest that FAP expression can occur in both the stroma and epithelial compartments of cancers [30-33]. However, the selectivity of FAP for stromal fibroblasts, but not epithelial tumor cells, has been confirmed either by immunohistochemical studies in pancreas, colorectal and breast cancer patients [12,34,35], or by RT-PCR of pancreas, lung, and renal cell xenografts [36]. To confirm the putative significance of studying FAP-expression effects in stromal matrices, we first examined FAP expression in the human pancreatic cancer patient tissues and mouse xenografts by immunohistochemistry. We observed that FAP is highly expressed on the stromal fibroblasts of human pancreatic cancer, while it is undetectable both in the epithelial cancer cells (Additional file 1, Fig. S1A) and normal tissue (not shown). In xenograft mouse models inoculating the human pancreatic cancer cell lines (HPAF-II, Capan-1, AsPC-1, and Panc-1), murine FAP expression was also found up-regulated specifically at the tumor stroma (Additional file 1, Fig. S1B). This observation confirmed the existence of a selectivity of FAP expression in tumor-associated fibroblasts and prompted us to believe that perhaps FAP is an attractive protein to study stromal effects imparted upon tumor behaviors.

### Stable FAP expression on naive fibroblasts

*In vivo*, FAP is highly expressed on pancreatic tumor stromal fibroblasts, but its expression *ex vivo *on cultured primary cells is not maintained. To establish stable FAP-expressing fibroblasts, mouse *fap *gene was cloned under the Tet-inducible system. Induction of FAP expression was achieved as early as 24 hours following Dox treatment, and its expression was clearly maintained for at least 10 days (Figure 1A). Parental NIH-3T3 cells and FAP^+ ^fibroblasts cultured in the absence of Dox showed no detectable FAP induction, allowing the use of these fibroblasts as negative controls.

### FAP expression on fibroblasts during 3D matrix production induces tumor stromal-like parallel orientation of fibronectin and collagen I fibers

During the 8 days required to produce a fibroblast-derived 3D matrix, clear morphological differences on fibroblasts were observed between the parental NIH-3T3 and FAP^- ^vs. FAP^+ ^fibroblasts. FAP^+ ^cells were elongated into an enhanced spindled shape, and they organized in a parallel pattern (data not shown). Since tumor-associated ECMs are organized in parallel patterns [21,22,37], we tested whether FAP expression affects the topography of fibroblasts-derived ECMs by indirect immunofluorescence using anti-fibronectin (Figure 1) or anti-collagen I (Additional file 2, Fig. S2) antibodies on un-extracted matrices containing their original matrix-synthesizing fibroblasts [21]. Indeed, FAP^+ ^fibroblasts produced ECM fibers oriented in parallel patterns when compared to FAP^- ^matrices (Figure 1B). These patterns are reminiscent of previously observed tumor-associated patterns *in vitro *[21]. In addition, the level of organization of the assorted fibroblast-produced fibronectin fibers was quantified by measuring the relative orientation angles of fibers [21]. The average percent of parallel fibers that were oriented within ±10^0 ^of the mode angle was determined using the MetaMorph software. The measured percentages were 27% and 45% for FAP^- ^and FAP^+ ^3D ECMs, respectively (p < 0.001) (Figure 1B). Furthermore, inhibition of FAP enzymatic activity by the FAP inhibitor during the matrix production effectively reversed the FAP-induced parallel matrix orientation (29%). Moreover, the observed architectural patterns were similar to the ones seen in desmoplastic reactions in human pancreatic cancers and in xenografted tumors formed in mice using human pancreatic cancer cells *in vivo *(see asterisks in Additional file 1, Fig. S1). Importantly, when assessing the architectural patterns of collagen I fibers using the FAP series of un-extracted matrices *in vitro*, our results showed that, although the collagen I fibrillogenesis levels during matrix production (8 days) were not as substantial as to allow the analysis of the fiber orientation, simple microscopy observations clearly demonstrated that FAP^+^ fibroblasts indeed affect collagen I fiber organization (Additional file 2, Fig. S2). These results suggest that the FAP enzymatic activity during matrix production is important for the topographical organization of the ECM fibers. Because of this topographical similarity to that of tumor permissive 3D ECMs [25,37], the FAP^+ ^3D matrix system was used to study the mechanism of matrix-supported pancreatic cancer cell invasion.

### Human pancreatic stellate cells- and adenocarcinoma-derived ECMs resemble FAP null and FAP^+^ ECMs, respectively

**Figure 2:**
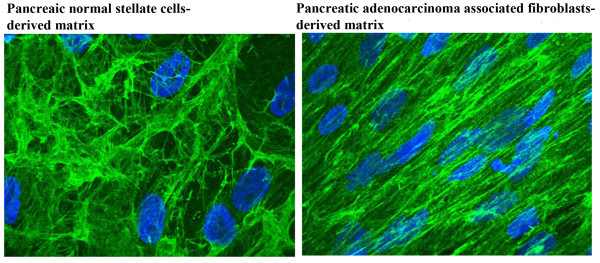
**Human pancreatic normal stellate cells and adenocarcinoma-derived ECMs resemble FAP^- ^and FAP^+ ^ECMs, respectively**. Human normal pancreatic stellate cells and pancreatic tumor-associated fibroblasts were isolated from fresh surgical tissue samples corresponding to matched (from the same case) normal pancreas (at least 2 cm away from the tumor) and pancreatic adenocarcinoma. Similar to images shown in Figure 1B, un-extracted human fibroblastic pancreas matrices (anti-fibronectin in green) were subjected to indirect immunofluorescence while cells' nuclei were labeled with DAPI (blue). Note how human pancreatic adenocarcinoma-associated fibroblasts produced ECMs oriented in parallel patterns, which are similar to the patterns formed by FAP^+ ^matrices while normal pancreatic stellate cells-derived matrices resemble FAP^- ^ECMs.

It is known that FAP exhibits very restricted expression in normal adult tissue [38] and that pancreatic stellate cells are the 'normal' fibroblastic cells found in the exocrine pancreas. Activated pancreatic stellate cells (i.e., myofibroblastic cells) express α-SMA and FAP (among other markers) and correspond to the cells believed to be responsible for the fibrosis in chronic pancreatitis and the desmoplastic reactions in pancreatic adenocarcinomas [39]. To compare our observations of the specific matrix architectures stemming from the FAP matrix series to matrices derived from normal pancreas human stellate cells and activated adenocarcinoma-associated fibroblasts, we proceeded to isolate these two types of human pancreatic fibroblastic cells from matched fresh surgical normal and pancreatic tumor (i.e., adenocarcinoma) samples (see Materials and Methods). Our results shown in Figure 2 present the standard bundle pattern of ECM fiber structures observed in FAP^- ^matrix. Moreover, human pancreatic adenocarcinoma-associated fibroblasts produced ECMs oriented in parallel patterns, which are similar to the patterns formed by FAP^+ ^matrices. This result supports the notion suggesting that FAP^+ ^fibroblast-derived matrices effectively recapitulate tumor stromal ECMs and that perhaps these matrices also have the capacity of producing matrices permissive/inductive of pancreatic tumorigenesis.

### Stromal FAP modulates the expression levels of assorted ECM proteins

**Figure 3:**
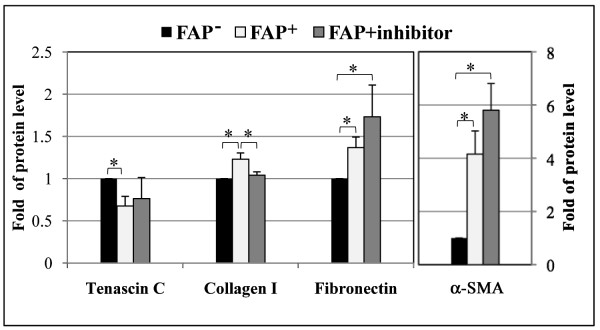
**FAP modulates the expression levels of stromal α-SMA and assorted ECM molecules**. Western analyses were used to determine the levels of desmoplastic marker α-SMA and assorted ECM proteins underlying the matrix differences mediated by FAP expression. The normalized fold ratios of the selected proteins by loading controls (β-actin or GAPDH) were assigned as one arbitrary unit. Compared to FAP^- ^matrix proteins, α-SMA, fibronectin, and collagen I were significantly up-regulated in FAP^+ ^3D matrices (p = 0.009, p = 0.002, p = 0.001, respectively), while tenascin C was significantly down-regulated (p = 0.001). Note that although collagen I levels were rescued (p = 0.007) by inhibition of FAP activity during matrix production (FAP+inhibitor), levels of expression of fibronectin and α-SMA were only significantly increased when compared to FAP^- ^(p = 0.04 in both cases) but not to FAP^+ ^(p = 0.32 and p = 0.61, respectively). *denotes statistical significance.

The ECM proteins and endogenous desmoplastic proteins (α-SMA) have been known to be up-regulated in pancreatic stromal reactions (desmoplasia) and play an active role in tumorigenesis [40]. The levels of ECM proteins (tenascin C, collagen I and fibronectin) and cellular α-SMA potentially underlying the matrix differences mediated by FAP were measured on the assorted un-extracted matrices (Figure 3). The expressions of α-SMA, fibronectin, and collagen I were significantly up-regulated in FAP^+ ^matrix compared to FAP^- ^matrix (p = 0.009, p = 0.002, p = 0.001, respectively), while tenascin C was significantly down-regulated in FAP^+ ^matrix (p = 0.001). Thus FAP expression in fibroblasts during matrix production alters ECMs composition, as well as its topography (e.g., fibronectin-fiber orientation). Interestingly, compared with FAP^-^, inhibition of FAP enzymatic activity during FAP^+ ^matrix production enhanced fibronectin and α-SMA expression (p = 0.04 and p = 0.04, respectively) to levels comparable to the ones observed in FAP^+^ cultures in the absence of the inhibitor. In fact, the P values for FAP^+ ^vs. FAP^+^inhibitor were 0.32 and 0.61 for fibronectin and α-SMA, respectively. On the other hand, blocking FAP activity during FAP^+ ^matrix production significantly down-regulated collagen I (p = 0.007), bringing the levels of this ECM protein back to levels similar to the ones observed in FAP^- ^matrix. These results suggest that collagen I production may be FAP activity dependent (Figure 3).

### FAP^+ ^3D matrices promote the invasiveness of pancreatic cancer cells

**Figure 4:**
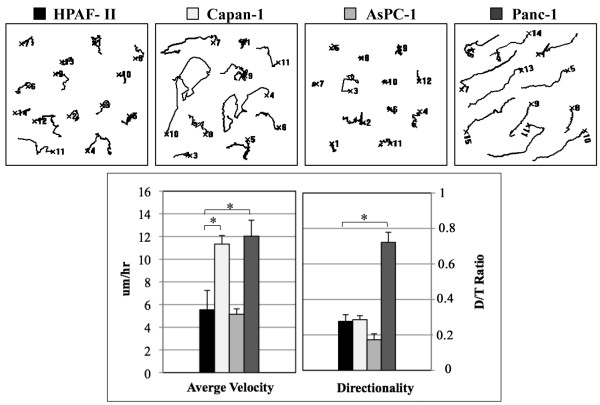
**Cell-dependent motility phenotypes were observed in FAP^+ ^matrix**. To determine ECM effects on the invasive behaviors of cancer cells, time-lapse acquisition assays were performed on FAP^+ ^matrices using pancreatic cancer cell lines. Representative tracks are shown where each cell line presented distinct responses to the FAP^+^ matrix as measured by average velocity (μm/hr) and directionality (D/T). Statistical significances were marked using asterisks.

To determine the effects of the ECM on the invasive behavior of cancer cells, time-lapse acquisition assays were performed using pancreatic cancer cell lines that are known for their distinct invasive and metastatic potentials *in vivo *[41]. The more metastatic cell lines (Capan-1, Panc-1) and the less aggressive cells (AsPC-1, HPAF-II) were plated onto matrices, and their behaviors were measured for average velocity (AV), net path distance (D), path trajectory (T), and directionality (D/T ratio) (Figure 4). The invasive behaviors of AsPC-1 (AV = 5.1 ± 0.7, D = 9.5 ± 2.9, T = 54.8 ± 5.0) and HPAF-II cells (AV = 5.5 ± 1.7, D = 16.6 ± 4.8, T = 58.1 ± 18.1) were found to be less effective than the behaviors of Capan-1 (AV = 11.3 ± 0.7, D = 32.2 ± 0.7, T = 120.8 ± 7.9) and Panc-1 cells (AV = 12.0 ± 1.4, D = 94.1 ± 13.1, T = 128.2 ± 15.0) within the FAP^+ ^matrix. The greater average velocity of Capan-1 and Panc-1 correlated with their known enhanced ability to metastasize *in vivo *[41]. Although Capan-1 and Panc-1 cells presented similar velocities, they showed significant differences in their movement patterns as Panc-1 cells invaded using greater directional motilities (D/T = 0.72 ± 0.05) compared to the more random paths taken by the Capan-1 cells (D/T = 0.28 ± 0.02, p < 0.001). However, no significant differences in velocity or directionality were observed using these cells cultured within the FAP^- ^matrices (not shown).

In order to question whether FAP^+^ matrix effects promoting cell motility of invasive pancreatic cancer cells (i.e., Panc-1) are specific for pancreatic cancers, we repeated this series of experiments using three well characterized breast cell lines. Additional file 3, Fig. S3 shows that compared to immortalized normal MCF-10A (AV = 7.59±1.3, D/T = 0.348±0.09) and tumorigenic MCF-7 (AV = 4.88±0.65, D/T = 0.17±0.042), highly invasive MDA-MB-231 cells moved faster (AV = 12.0±1.0, p = 0.04, p < 0.001, respectively) and more direct (D/T = 0.68±0.04, p = 0.001, p < 0.001, respectively) on FAP^+^ matrix. The results demonstrate that FAP^+^ matrix effects may be important in neoplasias other than just pancreatic cancers.

In summary, FAP^+ ^3D matrices represent a permissive environment for pancreatic (and breast cancer) invasion, and perhaps these matrices play an equally important role along with the epithelial cell component. Given their matrix-induced velocity and persistent directionality, Panc-1 (and in some instances MDA-MB-231) cells were selected for the further studies.

### FAP^+ ^3D matrices induce enhanced invasion behaviors upon Panc-1

**Figure 5:**
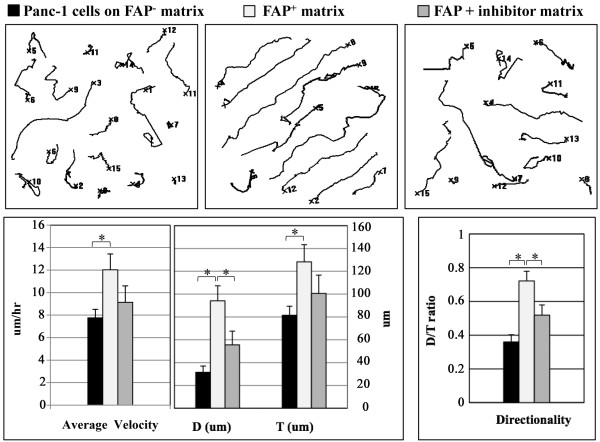
**FAP^+ ^ECMs promote a tumor permissive microenvironment**. To test the effects of FAP on the behavior of cancer cells, Panc-1 cells on assorted ECMs were monitored using time-lapse acquisition. Compared to their behavior within FAP^- ^matrix, Panc-1 cells presented increased velocity and directionality within FAP^+ ^matrix (p = 0.05 and p < 0.001, respectively). When compared to behaviors induced by FAP^+ ^matrices, Panc-1 cells on FAP+inhibitor matrix attenuated net path distances (D) and directionality (p = 0.07 and p = 0.01, respectively), but not velocity (p = 0.19). Statistical significances were marked using asterisks.

To test the contribution of FAP in the matrix-induced permissive tumor behavior, Panc-1 cells were seeded onto the three distinct matrices produced from the FAP^- ^, FAP^+^, or FAP+inhibitor fibroblasts (Figure 5). Compared to cells in FAP^- ^matrix (AV = 7.8 ± 0.8, D/T = 0.36 ± 0.04), Panc-1 cells presented greater velocity and directional motility within FAP^+ ^matrix (AV = 12.0 ± 1.4 and D/T = 0.72 ± 0.06) (p = 0.05 and p < 0.001, respectively). In addition, directionality was significantly attenuated on FAP+inhibitor matrix (D/T = 0.52 ± 0.06, p = 0.01), with a minor inhibition in the velocity (AV = 9.1 ± 1.6, p = 0.19). These results demonstrate that velocity and directionality of cells depend on the type of ECM, which can differ if the matrix-producing fibroblasts express FAP or not. In addition, these results suggest that while velocity is FAP dependent regardless of its activity, directionality is strictly dependent on FAP enzymatic activity.

### Matrix-mediated invasive phenotypes are regulated by β1-integrins

**Figure 6:**
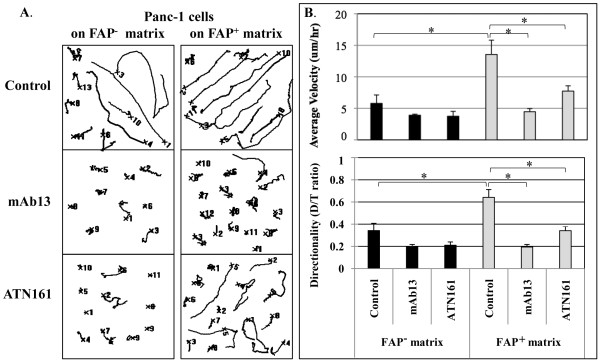
**Matrix-mediated Panc-1 invasive phenotypes are regulated by β_1_-integrin**. To determine the engagement of integrins for the invasiveness of Panc-1 in FAP^+^matrix, time-lapse acquisition assays were performed in the presence of functional blocking β_1_-integrin antibody mAb13 and blocking peptide ATN-161. (A) Panels are shown representative examples of migration tracks of individual cells invading through assorted matrices. (B) Distinct responses of Panc-1 cells to the integrin inhibition were measured by average velocity and directionality. Compared to the control experiment using rabbit serum, inhibition of β_1_-integrins significantly attenuated both velocity (67% inhibition, p < 0.001) and directionality (70% inhibition, p < 0.001) of Panc-1 cells in FAP^+ ^but not in FAP^-^matrix (p = 0.07 and p = 0.06, respectively). In addition, specific inhibition of α_5_β_1_-integrin decreased both velocity (43% inhibition, p = 0.002) and directionality (53% inhibition, p < 0.001) of Panc-1 cells on FAP^+ ^matrix. Statistical significances were marked using asterisks.

Integrins are the major receptors that link the ECM tumor microenvironment with the migrating cancer cell. Given the enhanced expression of ECM proteins and rearranged fibronectin fiber orientation in FAP^+ ^matrix, the contribution of integrins responsible for the invasiveness of Panc-1 on matrix was investigated by time-lapse acquisition assays performed in the presence of functional blocking agents, including the β_1_-integrin antibody mAb13 and the α_5_β_1_-integrin blocking peptide ATN-161 (Figure 6). Compared to the control experiment using rabbit serum (AV = 13.55 ± 2.25, D/T = 0.64 ± 0.07), inhibition of β_1_-integrins significantly attenuated both velocity (AV = 4.46 ± 0.49, 67% inhibition, p < 0.001) and directionality (D = 0.19 ± 0.03, 70% inhibition, p < 0.001) of Panc-1 cells on FAP^+ ^matrix. However, these significant inhibitions were not observed on cells using FAP^- ^matrix (p = 0.07 and p = 0.06, respectively). This result suggests that β_1_-integrin is an important regulator of tumor cell motility in FAP^+ ^but not FAP^- ^matrices. Similar to results above, questioning the matrix specific effect on only pancreas tumorigenesis, here we decided to test FAP^+^ matrix motility effects imparted upon MDA-MB-231 cells in the presence or absence of mAb-13. Results suggest that the observed FAP^+^ matrix induced increase in MDA-MB-231 motility is also dependent on β_1_-integrin activity (Additional file 4, Fig. S4).

Given the enhanced expression and spatial orientation of fibronectin in FAP^+ ^matrix, the importance of the fibronectin receptor α_5_β_1_-integrin was also tested. Inhibition of α_5_β_1_-integrin using ATN-161 showed significant decrease in the FAP^+ ^matrix-induced invasive behavior of Panc-1 cells (Figure 6). Compared with controls, inhibition of α_5_β_1_-integrin decreased both velocity (AV = 7.73 ± 0.84, 43% inhibition, p = 0.002) and directionality (D = 0.34 ± 0.03, 53% inhibition, p < 0.001) of cells on FAP^+ ^matrix, although not to the same extent as that of more general integrin inhibitors. Given the incomplete abrogation of invasive behavior of Panc-1 cells in the presence of ATN-161, it is possible that additional β_1_-integrins could also contribute to pancreatic cancer invasion facilitated by the FAP^+ ^3D matrices. In summary we concluded that, since the enhanced motility of Panc-1 cells on FAP^+ ^3D matrices can be significantly reversed by blocking the function of β_1_-integrins, a potential mechanism of matrix mediated tumor invasion is an attractive possibility.

### FAP^+^-dependent enhanced invasion behavior of Panc-1 is regulated by the β_1_-integrin/FAK signaling pathway

**Figure 7:**
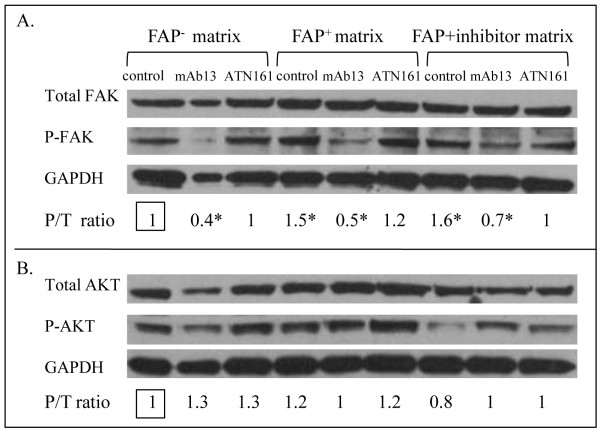
**FAP^+ ^matrix-induced Panc-1 invasion is dependent of FAK**. Panc-1 cells were cultured within the assorted matrices for 2 days in the presence of mAb13, ATN-161, or rabbit sera. The specific activity of the kinases was calculated as the ratio of the scanned optical densities of the phosphorylated forms divided by total expression levels (P/T). The fold of P/T ratios being assessed obtained from FAP^- ^matrices (square box) was assigned as one arbitrary unit. (A) Constitutive FAK activity levels of Panc-1 cells induced by FAP^+ ^matrix were up-regulated by ~1.5 fold (p = 0.007). Inhibition of β1-integrin activity significantly blocked the FAK activity levels of Panc-1 cells cultured within both FAP^- ^and FAP^+ ^matrices (p = 0.02 and p = 0.002, respectively). However, inhibition of α_5_β_1_-integrin showed no significant changes in FAK activity levels (all p > 0.3). (B) AKT activity in Panc-1 cells on matrices remained unchanged in the presence or absence of inhibitory agents. Statistical significances were marked using asterisks.

Integrin-mediated signaling pathways are often associated with AKT and/or FAK effectors, resulting in proliferation, survival, and invasion of tumor cells [42]. To determine the downstream components of β_1_-integrins, Panc-1 cells were cultured within assorted 3D matrices and the constitutive activity levels of both AKT and FAK were analyzed by the ratios of pS^473^-AKT/total AKT and pY^397^-FAK/total FAK, respectively (Figure 7). As shown in Figure 7A, constitutive FAK activity levels of Panc-1 cells cultured within both FAP^+ ^and FAP+inhibitor matrices were significantly up-regulated ~1.5 fold compared to its activity in FAP^- ^matrix (p = 0.007 and p = 0.03, respectively). When activity levels were compared for Panc-1 cells cultured within the assorted matrices in the presence or absence of mAb13, a significant down-regulation of constitutive FAK activity levels under β_1_-integrins inhibition was observed in all three matrices (p = 0.02, p = 0.002, p = 0.02 for FAP^-^, FAP^+^, FAP+inhibitor matrices, respectively). However, constitutive levels of AKT activity in Panc-1 cells cultured within the various matrices remained unchanged (all p > 0.18, Figure 7B). In addition, inhibition of α_5_β_1_-integrin with ATN-161 did not show any changes in the activities of FAK or AKT (all p > 0.2), suggesting that the specific integrin contributions to the total AKT and FAK activity levels are undetectable by this method, nevertheless, ATN-161 effects were shown in the motility assay (Figure 6). In summary, up-regulation of FAK activity in Panc-1 cells on FAP^+ ^matrix and down-regulation of its activity by function blocking β_1_-integrin suggest that the FAP^+^-dependent enhanced invasion behavior of Panc-1 is regulated by the β_1_-integrin/FAK signaling pathway, and although α_5_β_1_-integrin seems to play a role, additional β_1_-integrins may also participate in this regulatory process.

## Discussion

In this work, we provide evidence that FAP is important for remodeling a permissive stromal ECM that supports pancreatic (and perhaps also breast) cancer invasion *in vitro*. The experimental approach used to study the role of FAP in tumor invasion utilized an *in vivo*-like 3D matrix system that has been shown to effectively recapitulate stromal ECMs from various murine and human tissues [21,28,43]. Fibroblasts-derived 3D matrix system can avoid the flat surface of a tissue culture dish which impose an artificially rigid environment onto the cell [44]. On recent studies analyzing the direct effects of tumor stromal ECMs on cancer cell behavior, we demonstrated (among other things) that different breast cancer cell lines present distinct cell motility phenotypes when cultured within tumor-associated ECMs [37]. Here we also performed assays using the same breast cancer lines on our FAP^+ ^matrices and found that FAP^+ ^matrices effectively recapitulate many aspects of tumor-associated ECMs (data shown in supplemental material). Thus FAP^+ ^matrices were utilized as a stromal landscape to study matrix-induced pancreatic (and in some instances breast) cancer cell behaviors.

In this study, we observed that FAP^+ ^fibroblast-derived matrices presented higher organization levels of fibers when compared to FAP^- ^matrices. Importantly, we showed that FAP^+ ^matrices contain parallel fiber organization features that are reminiscent of tumor-associated ECMs of pancreatic desmoplastic tissues associated with pancreatic adenocarcinoma looking at human normal and tumor ECMs both *in vivo *and *in vitro*. The observed enhanced directionality and velocity of cancer cells invading through FAP^+ ^3D matrices was effectively reversed in matrices produced from FAP^+ ^fibroblasts in the presence of FAP enzymatic inhibitor. By determining alterations in collagen at the vicinity of tumors *in vivo*, it has been suggested that a strong relationship exists between both collagen density and matrix architectural organization and observed increased breast tumorigenesis [22]. Likewise, tumor-associated collagen features similar to the above-mentioned were observed by depleting FAP *in vivo *[45]. Interestingly, our human pancreatic adenocarcinoma-associated fibroblast ECMs, as well as our FAP^+ ^3D matrices *in vitro*, also presented the parallel organized patterns highlighted by the two studies above. These facts reinforce the notion that our *in vitro *models may be regarded as being highly relevant.

Our FAP+inhibitor matrices resembled FAP^- ^matrices with lower ECM levels of organization and supported relatively impeded tumor cell behaviors. We also report that inhibition of β_1_-integrins abrogates the FAP^+ ^ECM facilitated invasive capabilities of pancreatic and breast tumor cells, suggesting that a cell-matrix β_1_-integrin engagement is important for this FAP^+ ^matrix-dependent process. We elucidated a potential molecular mechanism underlying the enhanced pancreatic cancer cell motility mediated by FAP^+ ^matrix. We found that FAP remodels the extracellular matrices through alterations of ECM proteins levels, as well as through increased fibronectin fiber patterned orientation. FAP expression regulates tenascin C, collagen I, fibronectin and α-SMA expressions, all known to play important roles in pancreatic tumorigenesis [7,46]. FAP activity seems to be important for the positive collagen I and the negative tenascin C regulation, yet absence of FAP activity seems to significantly block collagen I while enhancing fibronectin and α-SMA levels of expression. Our results suggest that FAP enzymatic activity plays a role not only in regulating fiber orientation but also in regulating expression levels of collagen I. Moreover, we were able to distinguish among FAP expression and/or activity dependent functions in regulating stromal markers. When measuring the levels of collagen I expression, we observed that, while FAP activity is important to both fibronectin and collagen architectural fiber organization, this activity is mostly important for assessing collagen I as opposed to fibronectin (or α-SMA) levels of expression. In summary, we observed that both FAP expression and/or activity dependent functions can regulate ECMs compositions.

Interestingly, we learned that the velocity of the migrating cancer cells is dependent on FAP expression regardless of its activity, whereas the latter is crucial for directionality. The observed change in architecture and the levels of components of the ECM leads to enhanced ECM permissiveness, which facilitates pancreatic cancer invasiveness via β_1_-integrin engagement. Interestingly, our results using MDA-MB-231 cells were comparable to the ones observed with Panc-1, and thus we concluded that fibroblastic FAP-dependent matrix alterations and the importance of β_1_-integrin in the regulation of cancer cell motility are effects that are not necessarily restricted to pancreatic cancers and that additional cancers such as breast, where stromal FAP expression levels have been shown to be increased [47], show similar behaviors. Furthermore, FAP^+ ^matrix-induced regulatory molecules in cancer cells revealed that it is associated with an increased activation of FAK that is independent of AKT activity. Because β_1_-integrins are major receptors responsible for ECM assembly, these results suggest that FAP remodels ECM fibers to provide directional paths for pancreatic (and other cancer, i.e., breast) cells to engage β_1_-integrin/FAK for invasion. As a response to altered signals from the tumor stroma, cancer cell behavior seems to be influenced through patterning of the underlying matrix, resulting in track-dependent cellular migration. Conversely, the inhibition of α_5_β_1_-integrin function only partially affected FAP^+ ^matrix-induced Panc-1 invasion. This specific integrin inhibition did not significantly alter the constitutive FAK activation seen in Panc-1 cells cultured into FAP^+ ^matrices, suggesting that additional members of the β_1_-integrin family are also responsible for the matrix-induced effects. Importantly, the changes in the ECM produced by FAP^+ ^fibroblasts effectively recapitulate many aspects of stromal ECMs, including increased tumorigenic behaviors [37], enhance ability to metastasize *in vivo *[41], promote epithelial cancer cells to move along insoluble tumor-associated ECM fibers leading them towards the intravasation sites during metastasis [48], and recapitulate pancreatic stromal characteristics, such as increased collagen I expression and up-regulation of desmoplastic marker α-SMA [49].

## Conclusions

Our data suggests that cancer cell invasiveness can be affected by alterations in the tumor microenvironment. We provide evidence that FAP is important for remodeling a permissive stromal ECM to produce directional paths for pancreatic (and breast) cells that engage β_1_-integrin/FAK for invasion. Therefore, our observations imply that a better understanding of the changes in stromal fibroblasts and their influence on epithelial tumor cell behavior can lead to novel strategies for the prevention and treatment of cancer. The identification of fibroblastic 3D-dependent signaling pathways may be important to block the transition to a permissive microenvironment and even reverse stromal fibroblast-dependent neoplasia [44]. This study provides the pre-clinical rationale that inhibition of FAP proteolytic activity and selected β_1_-integrin family members in combination may abrogate the invasive capabilities of pancreatic tumors by interfering with the architectural organization of tumor-associated ECMs and disrupting the tumor-stromal interactions.

## Competing interests

The authors declare that they have no competing interests.

## Authors' contributions

HOL performed the majority of experiments and analyses and drafted the manuscript. SRM performed the analysis of matrix fiber orientation. JFB harvested the human pancreas stellate and adenocarcinoma-associated fibroblasts, produced the human *in vitro *matrices, and performed the characterization analyses of these 3D ECMs. MV generated the Tet-inducible fibroblasts and provided insight while revising the manuscript. EC and JDK were responsible for the study coordination, as well as the design and interpretation of results and revision of the drafted manuscript. All authors read and approved the final manuscript.

## Pre-publication history

The pre-publication history for this paper can be accessed here:

http://www.biomedcentral.com/1471-2407/11/245/prepub

## Supplementary Material

Additional file 1 **Figure S1; FAP is selectively overexpressed in tumor stromal fibroblasts**. (A) In human pancreatic cancer patient samples, FAP is highly expressed in stromal fibroblasts (red arrows) but not in epithelial tumor components (black arrows) [18]. A parallel patterned ECM organization (*) is observed within the desmoplastic stromal reaction. (B) In a xenograft mouse model, human pancreatic cancer cell lines (HPAF-II, Capan-1, AsPC-1, and Panc-1) also induced murine FAP expression in the tumor stroma but not in tumor cells. Hematoxylin (blue) was used for counterstaining. Click here for file

Additional file 2 **Figure S2; FAP^+ ^fibroblasts affect the architectural patterns of collagen I fiber organization**. Un-extracted matrices were subjected to indirect immunofluorescence using a collagen I antibody (green) and their nuclei stained using DAPI (blue). Note FAP^+ ^fibroblasts produced ECM fibers with enhanced parallel patterns compared to both FAP^- ^and FAP+inhibitor matrices. Click here for file

Additional file 3 **Figure S3; A cell type dependent motility is observed in FAP^+ ^matrices using immortalized and neoplastic breast cell lines**. To test the general effect of FAP^+^matrices in promoting motility of invasive cancer cells, three human breast cell lines (immortalized normal MCF-10, tumorigenic MCF-7, and invasive MDA-MB-23) were assessed. Note that compared to MCF-10A and MCF-7, invasive MDA-MB-231 cells presented a faster (p = 0.04, p < 0.001, respectively) and more direct (p = 0.001, p < 0.001, respectively) motility within these permissive 3D matrices. Statistical significances were marked using asterisks. Click here for file

Additional file 4 **Figure S4; Matrix-mediated MDA-MB-231 invasive phenotypes are regulated by β_1_-integrin**. To confirm the engagement of integrins for the invasive characteristics of MDA-MB-231 on FAP^+^ matrix, time-lapse motility assays were performed in the presence of anti-integrin β_1 _antibody mAb13, the α_V_β_3 _specific integrin antibody LM609, and rabbit sera as a control. (A) Panels are shown representative examples of migration tracks of individual cells invading though assorted matrices. (B) Distinct responses of MDA-MB-231 cells to the integrin inhibition were measured by average velocity and directionality. Compared to the control, inhibition of β_1_-integrins significantly attenuated both velocity (p < 0.001) and directionality (p < 0.001) of cells in FAP^+ ^but not in FAP^- ^matrix. In addition, specific inhibition of α_V_β_3_-integrin decreased velocity (p < 0.001) but not directionality on FAP^+ ^matrix. Statistical significances were marked using asterisks. Click here for file
