# Supplementary figures and images for: FAP-overexpressing fibroblasts produce an extracellular matrix that enhances invasive velocity and directionality of pancreatic cancer cells
Source: BMC Cancer. 2011 Jun 13;11:245. doi: 10.1186/1471-2407-11-245 (PMC3141768; doi:10.1186/1471-2407-11-245)

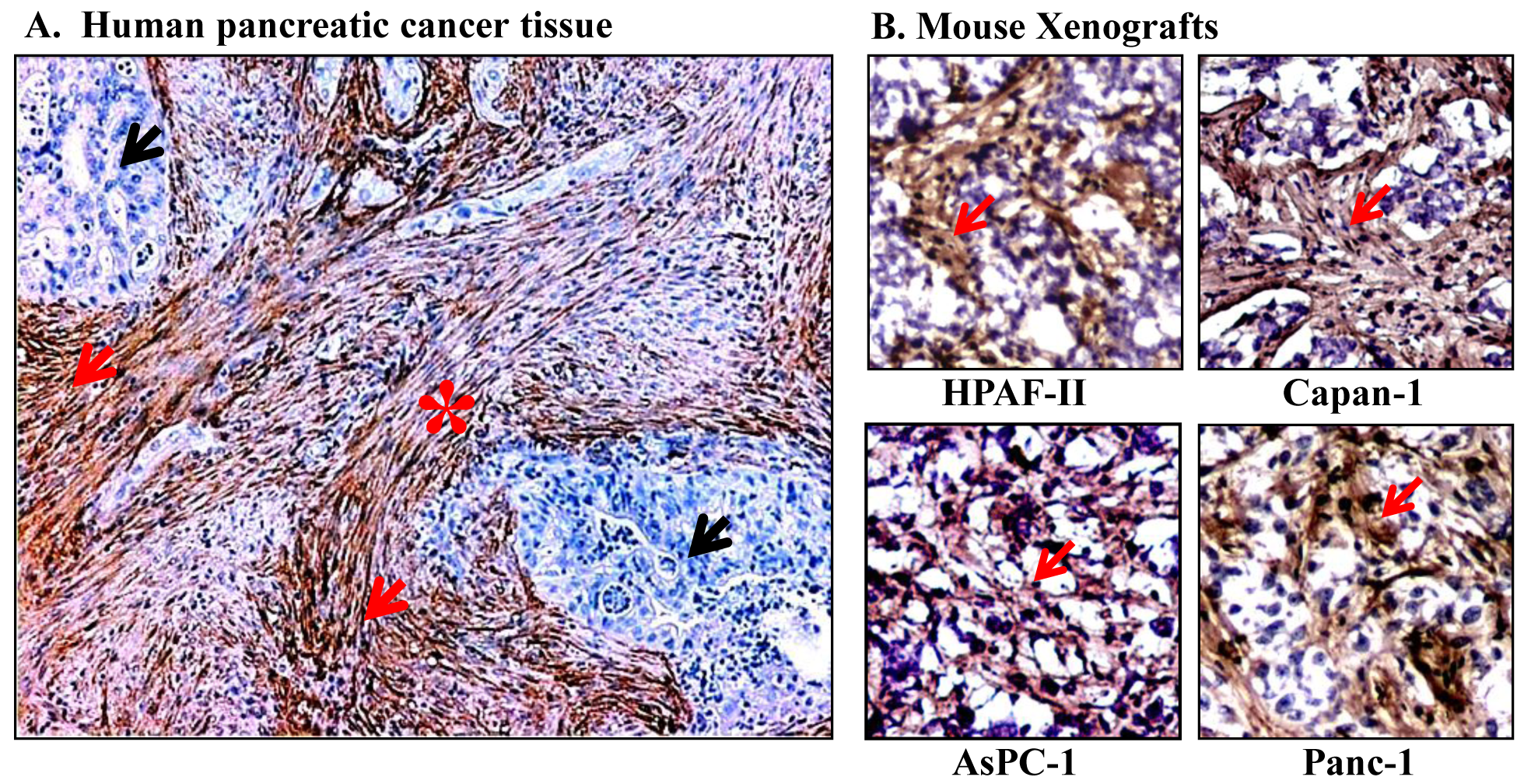

Supplement: Additional file 1 — Figure S1; FAP is selectively overexpressed in tumor stromal fibroblasts. (A) In human pancreatic cancer patient samples, FAP is highly expressed in stromal fibroblasts (red arrows) but not in epithelial tumor components (black arrows) [18]. A parallel patterned ECM organization (*) is observed within the desmoplastic stromal reaction. (B) In a xenograft mouse model, human pancreatic cancer cell lines (HPAF-II, Capan-1, AsPC-1, and Panc-1) also induced murine FAP expression in the tumor stroma but not in tumor cells. Hematoxylin (blue) was used for counterstaining. [file 1471-2407-11-245-S1.TIFF]

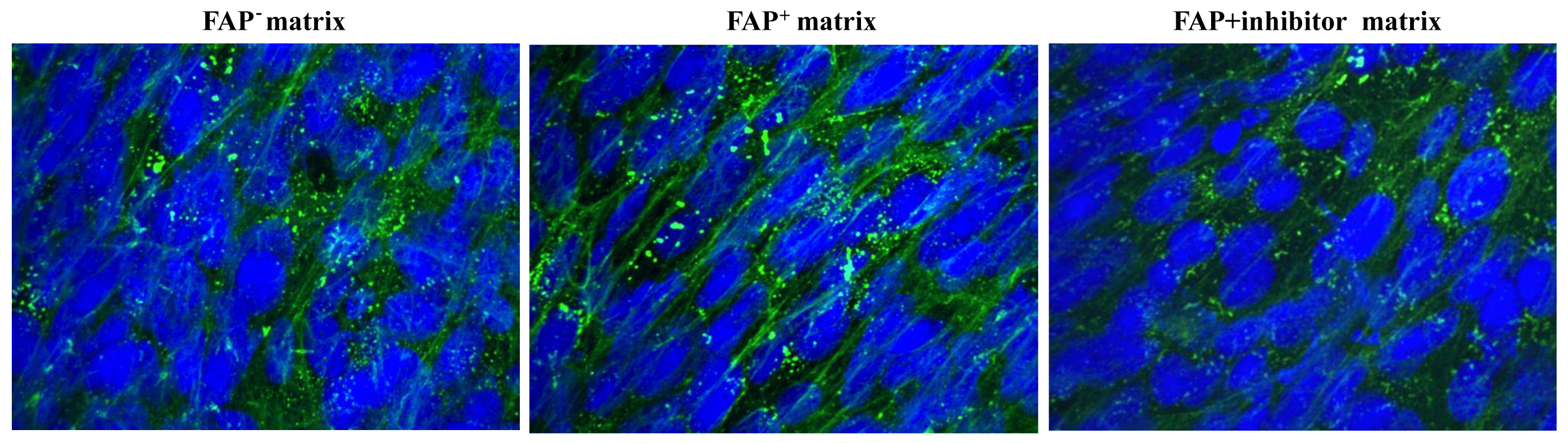

Supplement: Additional file 2 — Figure S2; FAP+ fibroblasts affect the architectural patterns of collagen I fiber organization. Un-extracted matrices were subjected to indirect immunofluorescence using a collagen I antibody (green) and their nuclei stained using DAPI (blue). Note FAP+ fibroblasts produced ECM fibers with enhanced parallel patterns compared to both FAP- and FAP+inhibitor matrices. [file 1471-2407-11-245-S2.TIFF]

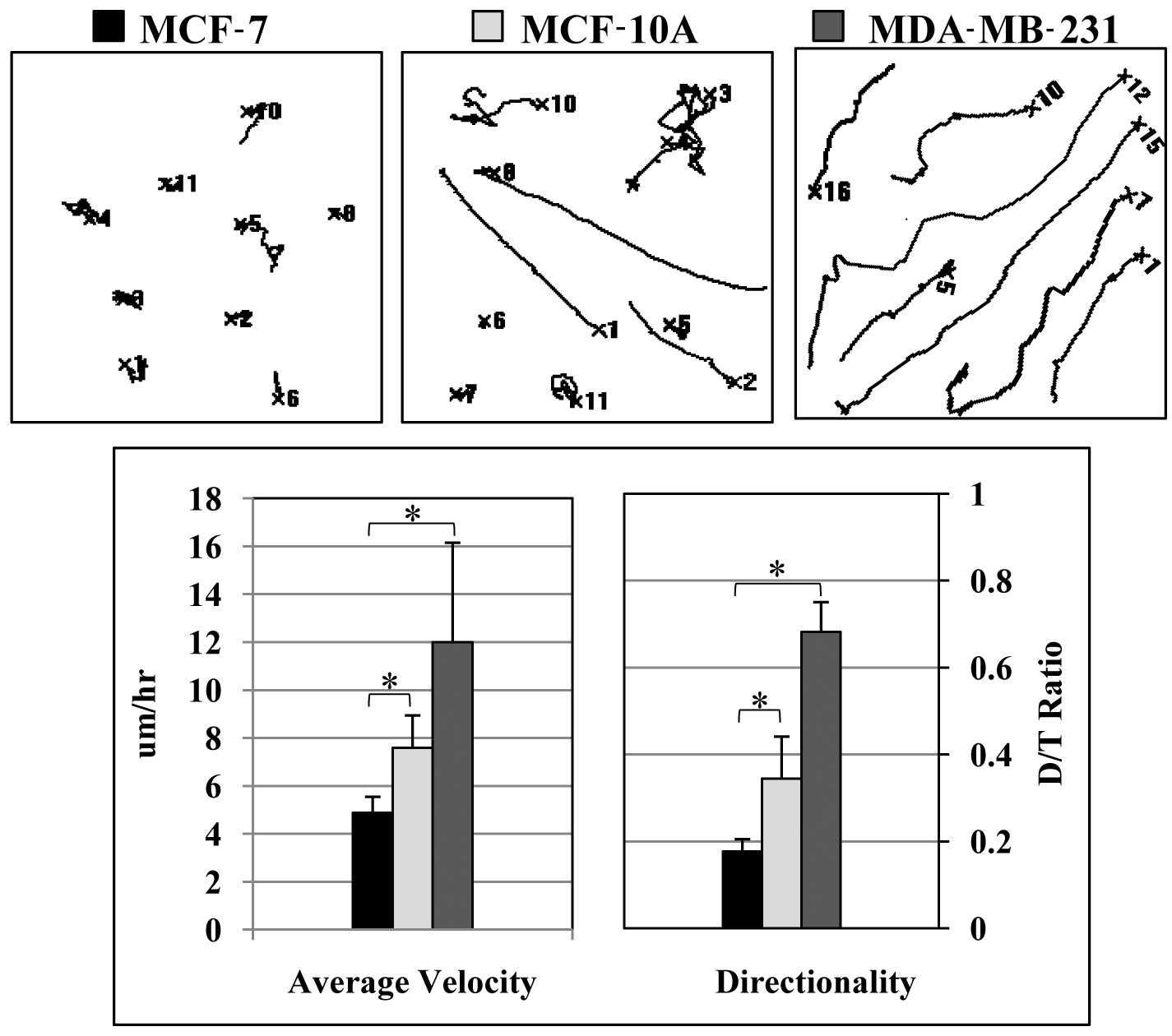

Supplement: Additional file 3 — Figure S3; A cell type dependent motility is observed in FAP+ matrices using immortalized and neoplastic breast cell lines. To test the general effect of FAP+matrices in promoting motility of invasive cancer cells, three human breast cell lines (immortalized normal MCF-10, tumorigenic MCF-7, and invasive MDA-MB-23) were assessed. Note that compared to MCF-10A and MCF-7, invasive MDA-MB-231 cells presented a faster (p = 0.04, p < 0.001, respectively) and more direct (p = 0.001, p < 0.001, respectively) motility within these permissive 3D matrices. Statistical significances were marked using asterisks. [file 1471-2407-11-245-S3.TIFF]

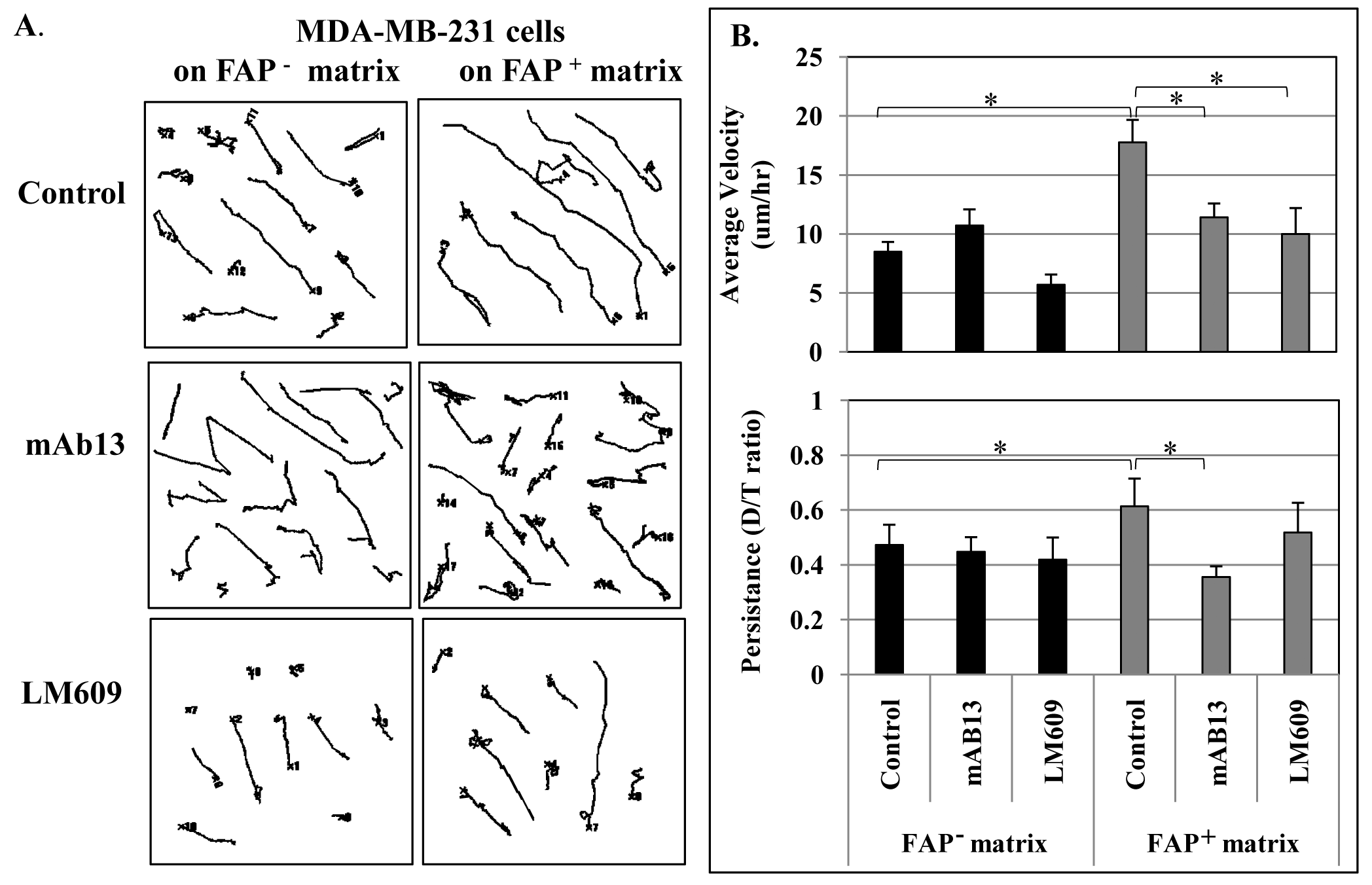

Supplement: Additional file 4 — Figure S4; Matrix-mediated MDA-MB-231 invasive phenotypes are regulated by β1-integrin. To confirm the engagement of integrins for the invasive characteristics of MDA-MB-231 on FAP+ matrix, time-lapse motility assays were performed in the presence of anti-integrin β1 antibody mAb13, the αVβ3 specific integrin antibody LM609, and rabbit sera as a control. (A) Panels are shown representative examples of migration tracks of individual cells invading though assorted matrices. (B) Distinct responses of MDA-MB-231 cells to the integrin inhibition were measured by average velocity and directionality. Compared to the control, inhibition of β1-integrins significantly attenuated both velocity (p < 0.001) and directionality (p < 0.001) of cells in FAP+ but not in FAP- matrix. In addition, specific inhibition of αVβ3-integrin decreased velocity (p < 0.001) but not directionality on FAP+ matrix. Statistical significances were marked using asterisks. [file 1471-2407-11-245-S4.TIFF]
